# An antimicrobial stewardship intervention on antibiotic prophylaxis for transrectal prostate biopsies in a veteran population

**DOI:** 10.1017/ash.2025.166

**Published:** 2025-07-07

**Authors:** Jessica Bennett, Tenley Ryan, Jane Eason, Neena Thomas-Gosain

**Affiliations:** 1 Lt. Col. Luke Weathers, Jr. VA Medical Center, Memphis, TN, USA; 2 University of Tennessee Health Science Center, Memphis, TN, USA; 3 University of Colorado School of Medicine, Aurora, CO, USA

## Abstract

This retrospective review describes changes in prophylactic antibiotic prescribing practices for Veterans undergoing transrectal ultrasound-guided prostate biopsy (TRUPB) and the incidence of post TRUPB infection-related hospitalizations before and after an antimicrobial stewardship intervention.

## Background

Prostate cancer is the most frequently diagnosed malignancy in men. For decades, transrectal ultrasound-guided prostate biopsy (TRUPB) has been the gold standard in diagnosing prostate cancer, but it is not without risk. Infectious complications have been reported in 1-6% of patients undergoing TRUPB^1^ including hospitalizations in 0.6 to 3%^2^ and life-threatening sepsis in 0.5 to 1.0%.^1^

Fluoroquinolones (FQ) were the mainstay for TRUPB antimicrobial prophylaxis (AP). Beginning in 2016, FDA labeling changes and growing data on increased FQ resistance and infectious complications following TRUPB led to guideline changes.^3–6^ Despite consensus in national guideline recommendations, use of AP in practice continues to vary and is often discordant with current guidance.^6–8^

Antimicrobial Stewardship (AS) has shown demonstrable impact on antimicrobial use, antimicrobial resistance, and clinical outcomes across healthcare settings. There have been few publications describing the role of AS in urology in the past decade, and the continued use of FQ and increasing rate of complications post TRUPB highlight the need for AS involvement.^7,9^

The purpose of this article is to describe an AS intervention on AP prescribing prior to TRUPB in a Veterans hospital and to evaluate differences in AP prescribing patterns before and after this AS intervention. Additionally, observed changes in infection-related hospitalizations (IRH) post-procedure will be described.

## Methods

### Intervention

In summer and fall of 2017, AS staff, composed of a part-time clinical pharmacist and consulting infectious disease physician, provided education via video chat to urology providers. Education included a review of updated American Urological Association (AUA) guidelines and local rates of infectious complications post TRUPB identified in a PRE-intervention review. The facility antibiograms, developed annually in accordance with Clinical and Laboratory Standards Institute guidelines^10^, include antimicrobial susceptibility rates for both inpatients and outpatients, and these trends were also included in the education. From October 2015 through September 2016 at this facility, 56%–81% of enteric gram-negative organisms were susceptible to ciprofloxacin, compared to 74%–87% susceptible to cefuroxime, and 80%–88% susceptible to ceftriaxone. The AS team recommended a change of AP for TRUPB from FQ to a 1^st^ or 2^nd^ generation cephalosporin given as a single dose based on AUA guidelines and local susceptibility patterns.^3^ Education was followed by short-term prospective audit and feedback by AS staff on prophylactic antibiotic prescriptions for TRUPB.

### Data and analysis

Records of all patients who underwent a TRUPB between June 1, 2016 and May 31, 2017 for the PRE-intervention review group (PRE) and October 2017 to May 2021 for the POST-intervention review (POST) were extracted from the Veterans Health Administration Corporate Data Warehouse (CDW). Procedures were excluded if the patient had a history of spinal cord injury, if an additional procedure occurred at the same time as TRUPB, or if TRUPB was aborted prior to completion. Demographic data, date of procedure and AP (including antibiotic choice, duration, and route) were procured from the CDW. Chart review was done to identify IRH within 30 days of TRUPB and confirm AP prescribed prior to the procedure and perioperatively. Identification of IRH related to TRUPB was based on documentation in the medical record of post-procedural GU infection (including cystitis, prostatitis, epididymitis, epididymo-orchitis) or bacteremia. Culture results related to the infection were collected if available.

The primary purpose was to describe differences in prescribing practices including AP route, choice, and duration before and after AS intervention. The rates of IRH within 30 days of TRUPB were also reviewed. Descriptive statistics and chi square analysis were used for categorical data with an alpha of 0.05 to determine significance. A student t-test was used to evaluate continuous data. Analysis was performed on a per-procedure basis.

## Results

For the PRE group, 272 TRUPBs performed on 263 patients were included for analysis. For the POST group, 588 TRUPBs performed on 533 patients were analyzed. For the majority of procedures, patients were prescribed an outpatient course of oral (PO) antibiotics to begin the day prior to the procedure. Additionally, patients may have received AP as PO, parenteral or combination of both perioperatively. Of note, 95 procedures (44 in PRE and 51 in POST) had a different PO antibiotic administered perioperatively than the antibiotic prescribed prior to the procedure. 98.2% (267/272) of the PRE group received oral-only AP, as compared to 51.9% (305/588) in the POST group (*P* < 0.05). Parenteral AP alone was not used for any procedure (Table 1). Regarding oral AP choice, there was a marked decrease in the use of ciprofloxacin between the PRE and POST groups (95.2% vs 9.9%; *P*< 0.05). Conversely, cefuroxime use significantly increased from the PRE to POST group (0.4% vs 93%; *P* < 0.05). Regarding parenteral choice, ciprofloxacin was the most prescribed parenteral AP (4/5, 80%) in the PRE group, compared to ceftriaxone (263/283, 93.3%) in the POST group. AP duration ranged from a single dose to 10 days in the PRE group and 1 to 6 days in the POST group. Average duration decreased from 3.4 days (SD+/-1.26 d) in the PRE to 3.0 days (SD+/- 0.38 d) in the POST group which was statistically significant (*P* < 0.05). IRH occurred after 10 of 272 (3.7%) TRUPB procedures in 10 unique patients in the PRE group compared to 5 of 588 (0.9%) procedures in 5 unique patients in the POST group (Table 2).

**Table 1.** Prophylactic antibiotics prescribed for transrectal ultrasound-guided prostate biopsy (TRUPB)

	No. Procedures with Patient Receiving at Least 1 Dose for TRUPB	
	PRE - Group n = 272	POST- Group n = 588
Oral **Antibiotics, No. (%)**		
Amoxicillin/clavulanic acid	0 (0)	15 (2.6)
Cefuroxime	1 (0.4)	547 (93)
Ciprofloxacin/Levofloxacin^+^	259 (95.2)	59 (10)
SMX/TMP	48 (17.6)	13 (2.2)
Other cephalosporin*	4 (1.5)	2 (0.3)
Other non-cephalosporin antibiotic**	0	2 (0.3)
**Parenteral Antibiotics, No. (%)**		
Cefazolin	0 (0)	1 (0.2)
Ceftriaxone	1 (0.4)	263 (44.7)
Ciprofloxacin	4 (1.5)	0 (0)
Gentamicin	0	19 (3.2)

Procedures may be associated with more than one antibiotic.

+ Levofloxacin n = 1 in POST-Group.

* Other cephalosporin includes: cephalexin, cefpodoxime.

** Other non-cephalosporin antibiotics: Fosfomycin, clindamycin.

**Table 2.** Characteristics of procedures complicated by infection-related hospitalization within 30 days

Procedure Characteristics	PRE - Group (N = 10)	POST - Group (N = 5)
**Patient age, yrs. (range)**	60.7 (48–69)	61.6 (53–70)
**Prior Positive Urine Culture, No. (%)**	1 (10%)	0
**Prior Exposure to Antibiotic Prescribed, No. (%)**	8 (80%)	0
**Infection, No. (%)**		
• *E.coli* bacteremia	5 (50%)	1 (20%)
• *Enterobacter aerogenes UTI*	0	1 (20%)
• *Klebsiella* bacteremia	1 (10%)	0
• Unknown organism	4 (40%)	3 (60%)
**Prophylactic Antibiotic Received. No. (%)**		
• Cefuroxime (PO) + Ceftriaxone (IM)	0	2 (40%)
• Cefuroxime (PO)	0	3 (60%)
• Ciprofloxacin (PO)	6 (60%)	0
• SMX/TMP (PO)	4 (40%)	0

## Discussion

This PRE- and POST-intervention review of AP prescribing for TRUPB identified discordance between consensus guidelines and local prescribing practices, a pattern previously described in the literature.^6,8,9^ This intervention answered the call by Augostini, *et al* for increased AS collaboration with urology resulting in optimization of antibiotic choice, namely an 85.3% decrease in FQ use (95.2 vs 9.9%).^8^ Additionally, a statistically significant decrease in antibiotic duration was observed POST-intervention. The average duration found in both groups was longer than the single dose recommended by AUA guidelines which highlights an area for continued intervention.^4^ Of interest, in our review, only 3 encounters in the PRE-group received single dose AP, none of whom had an IRH. No patients received single dose AP in the POST-group.

The incidence of IRH in both groups was consistent with rates reported in the literature.^1,2^ We observed a 75% risk reduction in IRH when comparing rates PRE- and POST-AS intervention. This finding is hypothesis generating as our study was not designed to measure the impact of an AS intervention on IRH and did not account for many confounding variables. We theorize that a change in antibiotic choice (from FQ to cephalosporins with more favorable local susceptibility patterns) in the POST group potentially contributed to a change in IRH rates.

This study had limitations due to its single-center retrospective design which included the potential for documentation errors and data collection bias. As previously mentioned, variables impacting IRH such as surgical risk factors were not collected and limit the ability to draw definitive conclusions. Finally, limited access to medical records outside our facility may have resulted in underestimation of IRH.

The positive changes in antibiotic choice and duration observed after AS intervention focused on AP for TRUPB were sustained for multiple years (2017–2021). This review describes an approach to AS intervention in Urology that utilizes consensus guidelines and local data and sheds light on future directions for study and intervention.
